# The role of antibody-drug conjugates in the treatment of lung cancer

**DOI:** 10.3389/fonc.2026.1757564

**Published:** 2026-02-05

**Authors:** Tanya Zlatanova, Aynura Changalova, Urska Janzic

**Affiliations:** 1 Clinic of Medical Oncology, Acibadem City Clinic Tokuda University Hospital, Sofia, Bulgaria; 2 Oncology center, Multi-profile Hospital for Active Treatment (MHAT) Uni Hospital, Panagyrishte, Bulgaria; 3 Medical Oncology Unit, University Clinic Golnik, Golnik, Slovenia; 4 Medical Faculty, University of Ljubljana, Ljubljana, Slovenia

**Keywords:** ADC, antibody-drug conjugate, lung cancer, non-small cell lung cancer, small-cell lung cancer

## Abstract

Over the las0t decades, lung cancer treatment has improved immensely, mainly due to the incorporation of new targeted treatments and immunotherapy. A relatively new and potentially highly effective class of drugs, antibody-drug conjugates (ADCs), has been introduced to the clinical setting and is currently under intense investigation, alone and in combination with other molecules. This study aims to summarize the latest data on ADCs for lung cancer treatment and to analyze their potential, toxicity profile, and challenges.

## 1 Introduction

The concept of the Magic Bullet, developed by Paul Ehrlich at the beginning of the 20th century (1), which aims to develop an ideal drug that targets harmful structures and spares normal tissues, was reintroduced decades later as the basis for the development of ADCs. It aimed to deliver a potent cytotoxic payload to cancer cells, thereby sparing normal tissues. The first ADC to enter the clinic was gemtuzumab ozogamicin, which was used to treat acute myeloid leukemia (2). After the initial approval by the US Food and Drug Administration in 2000, the drug was withdrawn due to safety concerns. However, it was re-approved in 2017 at lower doses and for specific patient populations.

In recent years, advances in antibody engineering, improved linker stability, and payload potency have led to the investigation and approval of many new ADCs for the treatment of various cancer types. Nevertheless, in lung cancer treatment, only a few ADCs have advanced to later clinical phases and entered guidelines. The role of ADCs in lung cancer remains poorly defined, and further evidence is needed.

With this review, we aimed to provide the audience with a deeper understanding of the mechanism of action of ADCs, their relationship to the specificity of the toxicity profile, current perspectives, and future directions of this class of anti-cancer drugs.

Methods: We searched PubMed and Google Scholar to identify relevant articles published from database inception to November 2, 2025, using the search terms “antibody–drug conjugates” and “lung cancer.” The review included articles published in English or translated into English; publications in other languages were excluded.

Inclusion and Exclusion Criteria.

Studies were included if they involved adult patients with lung cancer and reported original research with quantitative or qualitative data. Conference abstracts or study protocols were also included when results were available. Conference posters or non-peer reviewed data were not included.

Studies were excluded if they were non-human (animal or *in vitro*), involved populations outside the scope of the review (e.g., other cancer types or pediatric populations), or were letters, editorials, or commentaries without primary data. Case reports or small case series (<5 patients) were also excluded.

## 2 Key components of the ADCs and the mechanism of action

**Figure 1:**
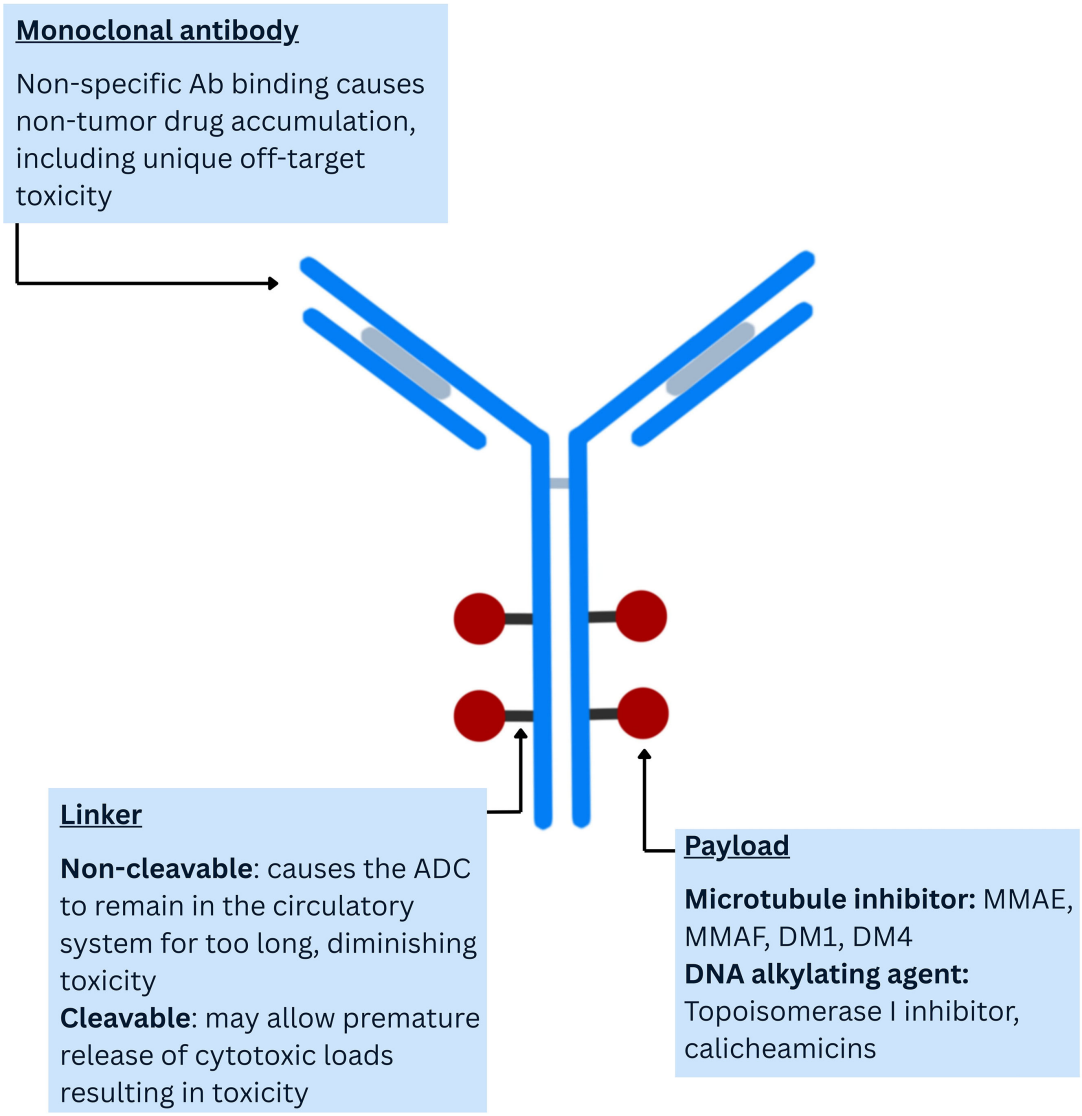
Structure of the ADCs.

The structure of the ADCs consists of a monoclonal antibody, a linker, and a cytotoxic payload (3, 4) (**Figure 1**).

The monoclonal antibody binds to a tumor-specific antigen expressed on the cell surface of tumor cells, and the payload attached to the antibody via the linker is then released within the tumor cell, leading to cancer cell apoptosis.

Different antigens have been recognized as appropriate targets for developing ADCs in lung cancer. Some of the tumor epitopes that became a relevant target and entered clinical research in non-small cell lung cancer (NSCLC) are the Trophoblast cell-surface antigen 2 (TROP2), Human epidermal growth factor receptor 2(HER2), Human epidermal growth factor receptor 3 (HER3), MET proto-oncogene receptor tyrosine kinase (MET), Nectin-4, Tissue factor, Epithelial growth factor receptor (EGFR), and Carcinoembryonic antigen-related cell adhesion molecule 5 (CECAM 5) (5, 6). On the other hand, fewer consistent cell-surface antigens were discovered for small-cell lung cancer (SCLC), the most investigated of which are TROP2, Seizure-related homolog 6 (SEZ6), Delta-like ligand 3(DLL3), and Cluster of Differentiation 276 (CD276), a transmembrane glycoprotein more commonly known as B7-H3 (7, 8). The most important features of a potential tumor-specific target would be overexpression on the tumor cell surface compared to healthy cells, positioning of the antigen-binding site on the cell membrane’s outer surface, the ability to internalize the ADC, and absence from systemic circulation (4).

The ideal antibody for an ADC should exhibit high binding affinity for the target antigen, low immunogenicity, high stability in the systemic circulation, rapid internalization, and a low molecular weight (4). The most commonly used antibody for ADCs in lung cancer is an IgG1 molecule, which has a serum half-life of 21 days, a high Fcγ receptor affinity, and acts as a potent immune effector (4). IgG1 is more effective at triggering antibody-dependent cell-mediated cytotoxicity (ADCC) and complement-dependent cytotoxicity (CDC) than other subclasses (4).

There are several features the ideal linker should possess; the most important is the ability to prevent antibodies from aggregating in the systemic circulation and to prevent premature release of the cytotoxic payload (4). There are two types of linkers – cleavable and non-cleavable: in cleavable linkers, the drug is released selectively in the cancer cell and causes cell death due to differences in pH, specific microenvironmental, or intracellular conditions of cancer cells compared to normal cells, while the drug release mechanism in non-cleavable linkers is due to lysosomal degradation (4).

The cytotoxic payload of the ADC is the drug’s warhead, which destroys cancer cells. It must be potent, soluble, stable in the systemic circulation, have lower immunogenicity, and have a small molecular weight (4, 5). The most widely investigated payloads are either microtubule inhibitors (for example, Emtansine, DM-1; Ravransine, DM-4; Vedotin, Monomethyl auristatin E (MMAE)) or topoisomerase I inhibitors (such as 7-ethyl-10-hydroxycamptothecin (SN-38), Deruxtecan (Dxd), etc.) (5, 9) (**Table 1**).

**Table 1:** Examples of some well investigated ADCs, their respective antibody and payload.

ADCs	Target antigen	Antibody	Linker	Payload	DAR
Ado-trastuzumab emtansine	HER2	Trastuzumab	Non-cleavable	DM-1 (Emtansine)	3.5
Trastuzumab deruxtecan			Cleavable	DXd (Deruxtecan)	8
Patritumab deruxtecan	HER3	Patritumab		DXd (Deruxtecan)	8
Sacituzumab govitecan	TROP2	Sacituzumab		SN-38 (Govitecan)	7.6
Datopotamab deruxtecan		Datopotamab		DXd (Deruxtecan)	4
Telisotuzumab vedotin	c-Met	Telisotuzumab		MMAE (Vedotin)	3.1
Tusamitamab ravtansine	CEACAM5	Tusamitamab	–	DM-4 (Ravtansine)	–

An important feature of ADCs is the drug-to-antibody ratio (DAR), which is the average number of cytotoxic molecules per antibody and ranges from 2 to 8 in current ADCs. DAR plays an important role in the efficacy and toxicity profile of the ADC. The higher the DAR, the more potent the ADC, but at the cost of greater toxicity. Trastuzumab deruxtecan, an ADC targeting HER2 in NSCLC, has a DAR of 8 and higher rates of hematologic toxicities (mainly neutropenia and anemia) and diarrhea, with grade (G) 3 or higher due to the cytotoxic payload, deruxtecan, a topoisomerase I inhibitor (10).

**Figure 2:**
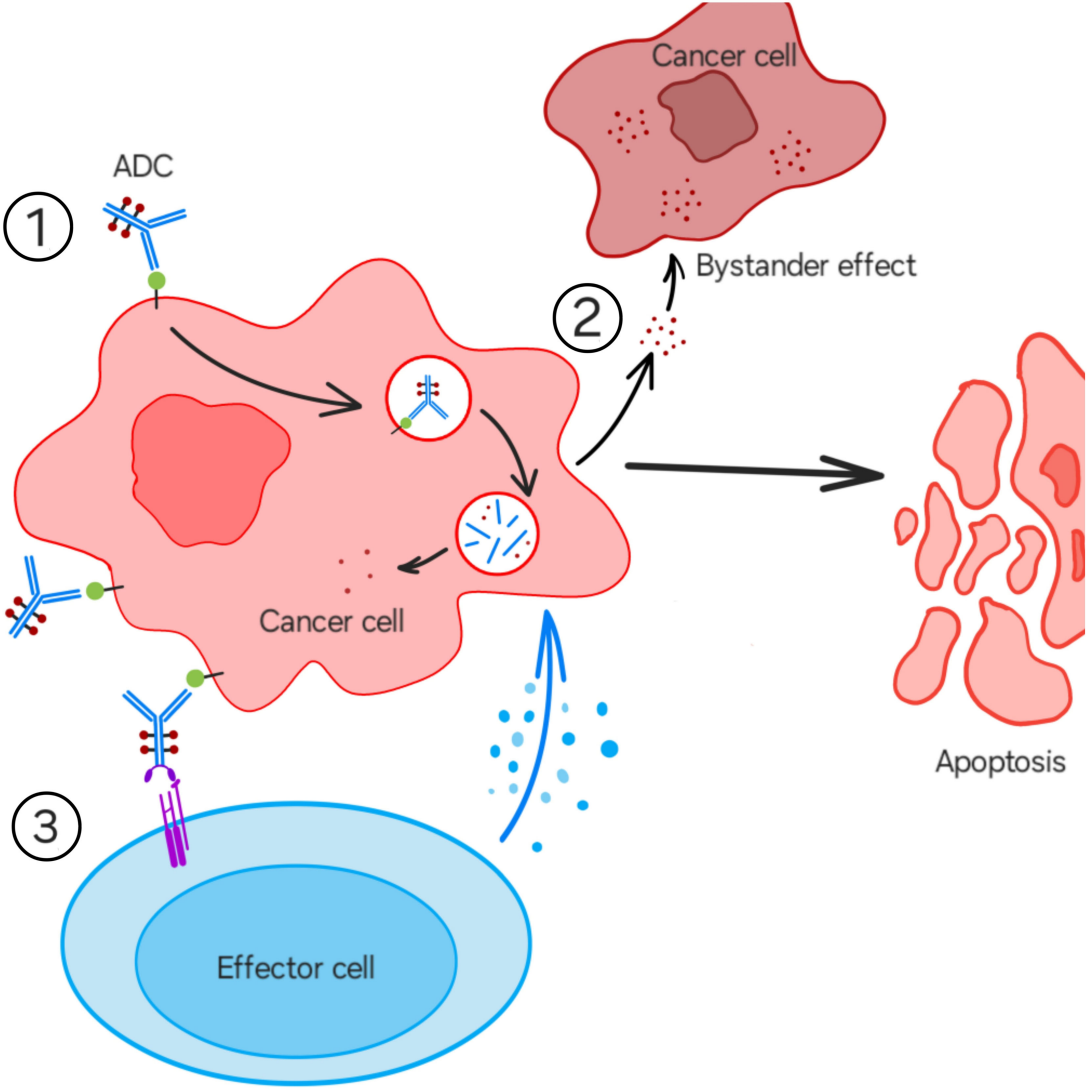
Mechanism of action of ADCs.

The ADC construct binds the specific antigen on the surface of the cancer cell, easily penetrates the cell membrane, and, after internalization and lysosomal degradation, the cytotoxic payload destroys the cancer cell by damaging its DNA in the tumor cell nucleus (**Figure 2**). Effector cells, such as natural killer (NK) cells, macrophages, and neutrophils, recognize the Fc region of antibodies via Fcγ receptors. When ADCs bind to tumor cell antigens, these effector cells destroy the tumor cells through antibody-dependent cellular cytotoxicity (ADCC) (**Figure 2**). Many ADCs also possess a complementary specific feature, known as a “bystander effect,” which is the ability of the released payload to penetrate through the cell membrane and enter the cancer cell’s microenvironment, having the ability to destroy adjacent cancer cells, even without having the expression of the specific antigen on their surface (**Figure 2**).

ADC characteristics that most strongly influence safety and efficacy include linker type, payload class, and drug-to-antibody ratio (**Table 2**).

**Table 2:** Key design features and toxicity signatures of antibody–drug conjugates (ADCs).

Feature	Category	Description/Characteristics	Clinical implications
Linker type 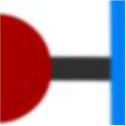	Cleavable linker	Sensitive to lysosomal enzymes, low pH, or reductive conditions; allows intracellular payload release and potential bystander effect	Enhanced tumor penetration; increased risk of off-target toxicity
	Non-cleavable linker	Requires complete lysosomal degradation of the antibody for payload release	Greater plasma stability; reduced bystander effect
Payload class (mechanism of action) 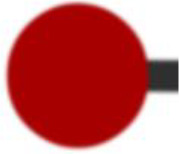	Microtubule inhibitors	Disrupt mitotic spindle formation (e.g., auristatins, maytansinoids)	Potent cytotoxicity; commonly associated with neuropathy
	DNA-damaging agents	Induce DNA strand breaks or inhibit DNA repair (e.g., calicheamicins, topoisomerase I inhibitors)	Effective against low-antigen tumors; higher hematologic toxicity
Drug-to-antibody ratio (DAR) 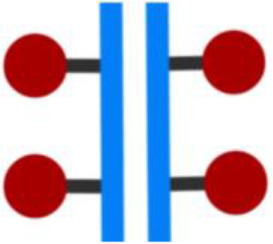	Low DAR (≈2)	Fewer payload molecules per antibody	Improved stability; potentially reduced efficacy
	Intermediate DAR (≈4)	Balanced payload loading	Optimal efficacy–toxicity balance in many approved ADCs
	High DAR (≥6–8)	Increased cytotoxic payload density	Enhanced potency; increased aggregation and systemic toxicity
Characteristic toxicity signatures	Hematologic toxicity	Neutropenia, anemia, thrombocytopenia	Common with DNA-damaging payloads
	Hepatotoxicity	Elevated transaminases, liver injury	Often related to off-target uptake
	Neurotoxicity	Peripheral neuropathy	Frequently associated with microtubule inhibitors
	Pulmonary toxicity	Interstitial lung disease/pneumonitis	Reported with specific ADCs and payloads

## 3 ADCs in SCLC

### 3.1 Targeting DLL3

The story of ACDs in lung cancer began in 2015 with the discovery of high delta-like ligand 3 (DLL3) expression on SCLC cell lines and tumor samples (11). Rovalpituzumab Tesirine (Rova-T) was investigated in the first-in-human, first-in-class, open-label phase I clinical study in recurrent small-cell lung cancer, which paved the way for this novel drug (12). Rova-T is a DLL3-targeted antibody-drug conjugate consisting of a humanized IgG1 antibody against DLL3 conjugated to the cytotoxic pyrrolobenzodiazepine (PBD) payload via a protease-cleavable linker. DLL3 is expressed in more than 80% of patients with SCLC (12) and is unlikely to be found in normal tissues, making it a good target for further investigation. The preliminary results of this study were encouraging, with an objective response rate (ORR) of 18% for Rova-T as monotherapy, particularly in tumors with high DLL3 expression (≥50% in tumor cells), and a manageable toxicity profile. Based on that, several phase I, II, and III studies were launched to investigate Rova-T, as monotherapy or in combination, in patients with extensive-stage SCLC (ES-SCLC) across different lines of treatment (13–16).

The phase III MERU trial compared Rova-T with placebo as a maintenance treatment in patients with SCLC after platinum-based chemotherapy, regardless of DLL3 expression (13). Unfortunately, the study did not meet the primary endpoints of improved progression-free survival (PFS) and overall survival (OS) in patients with DLL3-high tumors. Median OS was 8.5 months with Rova-T versus 9.8 months with placebo (hazard ratio (HR) 1.07; 95% confidence interval (CI): 0.84–1.36), meeting futility criteria; the study was terminated early for lack of OS benefit (13). Hence, the Central Radiographic Assessment Committee (CRAC) evaluation of PFS was not performed. Rova-T was linked to distinctive toxicities, including pleural and pericardial effusions, photosensitivity reactions, and peripheral edema (13).

Another phase III study was TAHOE, an open-label, two-to-one randomized trial comparing Rova-T with topotecan as second-line treatment in DLL3-high SCLC, with OS as the primary end point (14). Rova-T exhibited inferior OS and higher toxicity compared to the standard of care at that time, topotecan. Median OS was 6.3 months (95% CI: 5.6–7.3) with Rova-T and 8.6 months (95% CI: 7.7–10.1) with topotecan (HR 1.46; 95% CI: 1.17–1.82). Given the shorter OS in the Rova-T group, the independent data monitoring committee recommended stopping enrollment.

Later studies investigated Rova-T in combination with chemotherapy or different immunotherapies (namely nivolumab or nivolumab plus ipilimumab), but efficacy was limited, and toxicity was higher, leading to discontinuation of further drug development (15, 17). Furthermore, the phase II TRINITY study did not validate DLL3 expression as a reliable predictive biomarker for Rova-T (16), and subsequent studies with this drug did not use DLL3 levels for patient selection or stratification. The study included patients with DLL3-expressing SCLC and ≥ two prior regimens and demonstrated moderate clinical activity in 3L+ SCLC, with associated toxicities, including fatigue, photosensitivity reactions, and pleural effusions (16).

First-generation DLL3 ADCs used PBD dimer payloads, which are extremely potent DNA-crosslinking agents with a narrow therapeutic window. Despite the fact that early studies showed antitumor activity, systemic release of the payload led to severe off-target toxicities including pleural and pericardial effusions, edema and capillary leak- like syndromes, photosensitivity reactions, and hematologic toxicity. This severe toxicity was even amplified in the fragile patient population of patients with relapsed or refractory SCLC.

Although DLL3 is highly expressed in SCLC, DLL3 expression levels did not consistently correlate with clinical benefit in later-phase trials. Key issues included heterogeneous intratumoral expression, lack of validated, standardized cut-offs, and failure of DLL3 ICH to function as a reliable predictive biomarker. Nevertheless, DLL3 remains a desirable target for further investigation.

ZL-1310 is a novel ADC designed to target DLL3, utilizing the tumor microenvironment-activatable linker-payload (TMALIN) platform. It consists of a humanized anti-DLL3 antibody, a protease- cleavable linker (methylsulfonyl pyrimidine tripeptide), and a unique camptothecin-derived payload, which is also a topoisomerase I inhibitor. This ADC features a high DAR 8 with uniform conjugation and hydrophilic linker-payload combinations (18). ZL-1310 is being tested in patients with previously treated ES-SCLC who have received at least one prior platinum-based chemotherapy regimen, and preliminary results from the ongoing global phase Ia/Ib study of ZL-1310 were presented at the EORTC-NCI-AACR (ENA) Symposium 2024 in Barcelona, Spain (19). With an ORR of 74% (95%CI, 48.8 - 90.9) across all tested dose levels of ZL-1310 and a favorable pharmacokinetics (PK) and safety profile, ZL-1310 has the potential to deliver antitumor responses in the majority of patients with ES-SCLC (19). Based on these findings, on January 29, 2025, the U.S.FDA granted Orphan Drug Designation to ZL-1310 for the Treatment of small-cell lung Cancer.

Another DLL3-targeting ADC, SHR-4849, showed promising activity in a first-in-human phase 1 study (NCT06443489) at doses of 2.4 mg/kg or higher in patients with relapsed SCLC (20). The ORR was 73.2% (95% CI, 61.4%-83.1%) in all patients (n = 71), and the confirmed ORR was 47.9% (95% CI, 35.9%-60.1%). 92.0% of patients experienced any TRAEs, with 48.0% being G3 or higher (20).

Diverse current therapeutic approaches targeting DLL3 include ADCs, bispecific T-cell engagers (BiTEs), and chimeric antigen receptor (CAR) T-cell therapies (21).

### 3.2 Targeting B7-H3

Another ADC has shown promising results in heavily pretreated SCLC patients, as reported in a subset analysis of the DS7300-A-J101 trial presented at the International Association for the Study of Lung Cancer 2023 World Conference on Lung Cancer (22). B7 homolog 3 (B7-H3, CD276), a member of the B7 family of immune checkpoint proteins, interacts with multiple immune cells and influences various cellular processes, as it is expressed on both tumor cells and antigen-presenting cells (23). B7-H3 is highly expressed in SCLC and associated with poor prognosis. Ifinatamab derutxtecan (I-DXd, DS-7300), a B7-H3-directed antibody-drug conjugate, showed durable efficacy at ≥ 6.4 mg/kg (ORR, 52.4%; median duration of response (DOR) 5.9 months) in 21 patients with ES-SCLC in the Phase I/II IDeate-PT01 trial. The interim analysis (IA) results of the dose-optimization part of the ongoing Phase II IDeate-Lung01 study (NCT05280470) evaluating I-DXd in ES-SCLC demonstrated an ORR of 54.8% seen with I-DXd at a 12 mg/kg dose in pretreated patients (24). That is why 12 mg/kg was selected as the optimal dose for the extension part of the IDeate-Lung01 phase II trial, and the IDeate-Lung02 phase III study was recently initiated.

HS 20093 (GSK5764227, GSK’227) is an ADC with a fully humanized IgG1 that is linked via a cleavable maleimide tetrapeptide linker to a topoisomerase I inhibitor that is a derivative of exatecan - HS 20093 targets B7-H3, with a DAR4 targeted payload. In the ARTEMIS-001 study (NCT05276609), a multicenter, open-label phase I study investigating the safety and efficacy of HS-20093 in advanced solid tumors, patients with previously treated ES-SCLC received HS-20093 at 8 mg/kg every 3 weeks (n = 31). They achieved an ORR of 61.3% (95% CI, 42.2%-78.2%) (25). The disease control rate (DCR) was 80.6% (95% CI, 62.5%-92.5%) for the 8.0 mg/kg cohort. The B7-H3 biomarker analysis demonstrated low correlation between tumor B7-H3 expression and tumor objective response. In 2024, the FDA granted Breakthrough Therapy Designation to HS-20093 for ES-SCLC patients with disease progression after platinum-based chemotherapy, and China’s NMPA listed HS-20093 as a Breakthrough-Therapy-Designated Drug for ES-SCLC following first-line platinum-doublet chemotherapy and immunotherapy. On December 16, 2024, HS-20093 received EMA Priority Medicines (PRIME) Designation in relapsed ES-SCLC.

YL201 is another B7-H3-targeting ADC with a tumor microenvironment-activable linker and a novel topoisomerase I inhibitor payload. In a phase I study, 312 patients were enrolled across multiple tumor types, among them 79 patients with ES-SCLC were included who had progressed on one or two standard lines (26). Among the response-evaluable patients in this cohort, the ORR was 63.9% and the DCR was 91.7% (26). In patients with wild-type lung adenocarcinoma, the ORR was 28.6% (26). No significant correlation between B7H3 membrane expression and the ORR was found. Based on these encouraging results, a phase III study of YL201 in relapsed SCLC with progression on or after first-line platinum-based therapy was initiated (NCT06612151).

MHB088C, a novel B7-H3-targeted ADC containing a potent SuperTopo I payload, was investigated in patients with relapsed ES-SCLC in a phase 1/2 multicenter study (27). The latest results on efficacy and safety (ASCO 2025, Abstract #8510) show that ORR were 42.9%, 57.6%, and 46.7% in the 1.6, 2.0, and 2.4 mg/kg cohorts, respectively, with median PFS of 5.5, 5.9, and 5.5 months. No G5 toxicities were reported, and the most common G≥3 treatment-related adverse events were neutropenia, decreased platelet count, and anemia (27).

### 3.3 Targeting TROP-2

Sacituzumab govitecan (SG) is a TROP-2-directed ADC currently utilized in breast cancer and bladder cancer. However, TROP-2 is also expressed on SCLC and NSCLC epithelial cells and represents an attractive target in these tumor locations as well. The phase II TROPiCS-03 (NCT03964727) multicohort, open-label, basket study of solid tumors, including ES-SCLC, evaluated the efficacy/safety of SG as second-line treatment in patients with previously treated ES-SCLC (28). The primary endpoint of the study was investigator-assessed ORR, and among the 43 patients included, the ORR was 41.9% (95% CI: 27.0%–57.9%), with 18 confirmed partial responses (28). The authors concluded that SG has promising efficacy as a second-line treatment of ES-SCLC, irrespective of platinum sensitivity.

### 3.4 Targeting SEZ6

SEZ6 is a transmembrane protein expressed in SCLC and other neuroendocrine neoplasms (NENs), and central nervous system (CNS) tumors. In the phase I, open-label, multicenter, dose escalation and dose-expansion study (NCT05599984) ABBV-706, an ADC targeting SEZ6, conjugated to a topoisomerase I inhibitor payload at a DAR of 6, was investigated as monotherapy or in combination with budigalimab (a programmed cell death 1 inhibitor), carboplatin, or cisplatin for the treatment of SCLC, high-grade CNS tumors and high-grade neuroendocrine carcinomas (NECs) (29). The confirmed ORR was 21% (7 partial responses [PR]), 40% (6/15) for SCLC, and 6% (1/18) for NEN (29). Among 22 patients with SCLC and 22 with NENs, ABBV-706 demonstrated a manageable safety profile and promising efficacy, and further investigation is ongoing (29).

ABBV-011, a SEZ6-targeted antibody conjugated to calicheamicin, was evaluated in a first-in-human phase I study (NCT03639194) in patients with relapsed/refractory SCLC. In this study, 99 patients received ABBV-011 monotherapy; the drug was administered intravenously once every 3 weeks during dose escalation (0.3–2 mg/kg) and expansion. In the 1-mg/kg dose-expansion cohort (n = 40), the ORR was 25%, the drug was well tolerated, and showed promising activity in heavily pretreated patients with relapsed/refractory SCLC (30).

Despite the evidence above for the activity of some ADCs, no ADC has been approved by the FDA or EMA for the treatment of SCLC. While research into ADCs for SCLC continues, no ADC has yet demonstrated the efficacy and safety required for regulatory approval or inclusion in major clinical guidelines.

## 4 ADCs in NSCLC

### 4.1 Targeting HER2

Trastuzumab deruxtecan (T-DXd), an ADC targeting HER2, was investigated in the DESTINY-Lung01 phase II study in patients with metastatic HER2-mutant NSCLC who were refractory to standard treatment at a dose of 6.4 mg per kilogram of body weight (10). Among 91 patients with metastatic HER2-positive NSCLC who had progressed on 1st-line therapy, ORR was 55% (95% CI, 44 to 65). Of note, drug-related interstitial lung disease (ILD) occurred in 26% of patients and resulted in death in 2 patients (10). This led to the initiation of the DESTINY-Lung02 trial comparing T-DXd at a lower dose of 5.4 mg/kg once every 3 weeks in previously treated (platinum-containing therapy) patients, versus T-DXd at 6.4 mg/kg once every 3 weeks in this population (31). Confirmed ORR was 49.0% and 56.0% with 5.4 and 6.4 mg/kg, respectively (31). T-DXd is the only ADC currently approved by the FDA and EMA for the second-line treatment of NSCLC harboring a HER2 mutation.

### 4.2 Targeting HER3

Patritumab deruxtecan (HER3-DXd) is a HER3-targeting ADC that was investigated in patients with advanced EGFR-mutant tumors who had disease progression after EGFR tyrosine kinase inhibitor (TKI) therapy (amended protocol required prior osimertinib) and platinum-based chemotherapy (PBC) (32). In the phase II HERTHENA – Lung01 study, Patritumab deruxtecan achieved a confirmed ORR of 29.8%. In patients with untreated brain metastases, a substantial intracranial confirmed ORR of 33.3% and intracranial DCR of 76.7% were observed (32). Patritumab deruxtecan consists of a fully human anti-HER3 IgG1 monoclonal antibody, linked via a stable tetrapeptide-based cleavable linker to the cytotoxic payload exatecan derivative (a topoisomerase I inhibitor). HERTHENA-Lung02 (NCT05338970) is the first phase III trial in EGFR-mutated NSCLC after progression on an EGFR TKI, with results expected soon. In a press release issued by Daiichi Sankyo and Merck in September 2024, it was announced that the trial met its primary endpoint, demonstrating a statistically significant improvement in progression-free survival for patients treated with patritumab deruxtecan compared to those receiving platinum-based chemotherapy. The OS data were immature at that time, and the trial is ongoing to assess this secondary endpoint further.

### 4.3 Targeting TROP-2

Datopotamab deruxtecan consists of an anti-TROP2 IgG1 antibody, connected with a cleavable linker to the payload Deruxtecan derivate with a DAR of 4, and was investigated in the TROPION-Lung01 phase III study (NCT04656652), which included patients with advanced NSCLC (stage IIIB, IIIC, and IV) (33). Both patients with and without actionable genomic alterations (AGAs) were included, and Dato-Dxd at a dose of 6 mg/kg i.v. was administered Q3W and was compared to docetaxel 75mg/kg i.v. Q3W. In this study, Dato-Dxd significantly improved PFS compared to docetaxel, the standard of care, for patients with locally advanced or metastatic NSCLC treated in the second line setting with ORR 26.4% (21.5-31.8) vs. 12.8% (9.3-17.1), PFS HR 0.75 (0.62-0.91) (33). The results were better in the prespecified nonsquamous histology subgroup, where the median PFS was 5.5 vs. 3.6 months (HR, 0.63 [95% CI, 0.51 to 0.79]). However, these results did not translate into an OS improvement, and the median OS in the ITT population was 12.9 months with Dato-Dxd vs. 11.8 months with docetaxel (HR 0.94 [0.78-1.14]; p-value 0.530) (33). The phase II study TROPION-Lung05 investigated Dato-Dxd in patients with advanced or metastatic NSCLC harboring actionable genomic alterations (34). The confirmed ORR in patients with EGFR mutations was 43.6% (95% CI, 32.4 to 55.3) (34).

Datopotamab deruxtecan dlnk has been granted accelerated approval by the U.S.FDA for adult patients with locally advanced or metastatic, EGFR-mutated NSCLC who have received prior EGFR-directed therapy and platinum-based chemotherapy, based on pooled data (e.g., from the TROPION Lung01 and TROPION Lung05 trials) showing an ORR of around ~45% and a median duration of response of ~6.5 months in the EGFR-mutated subgroup.

Sacituzumab govitecan (SG), another anti-TROP2 ADC consisting of an anti-TROP2 IgG1 kappa antibody coupled to SN-38 via a cleavable linker, was investigated in a phase III study with a similar design. In the EVOKE-01 open-label study, SG was compared to docetaxel in the second-line setting in patients with metastatic NSCLC with or without AGAs (35). Although the primary end point of OS was not met, a numerical improvement in OS with SG vs. docetaxel was reported, consistent across histologies (35). The EVOKE-02 study (NCT05186974) investigated SG in combination with pembrolizumab, a PD-1–directed immunotherapy, with or without platinum chemotherapy, in first-line metastatic NSCLC, aiming to improve responses, particularly in patients for whom immunotherapy monotherapy may be insufficient (36). SG + pembrolizumab showed promising activity across histologies (squamous and nonsquamous) in previously untreated mNSCLC (36). In patients with PD-L1 ≥ 50% from Cohort A of the EVOKE-02 study, SG + pembro demonstrated promising activity, with an ORR of 67% (95% CI, 47-83%) in both squamous and nonsquamous histologies (37). The ongoing open-label multicenter phase III EVOKE-03 study compares the effectiveness of the combination of SG + pembrolizumab to pembrolizumab alone as first-line treatment in patients with PD-L1 TPS> 50% (NCT05609968) (38). Sacituzumab tirumotecan (Sac-TMT) consists of an anti-TROP2 IgG monoclonal antibody conjugated to a belotecan derivative with a novel linker, a topoisomerase I inhibitor, with a DAR of 7.4. In a recent non-randomized phase II OptiTROP-Lung01 study (NCT05351788), Sac-TMT was investigated in combination with KL-A167 (an anti-PD-L1 monoclonal antibody) as first-line treatment for patients with advanced NSCLC without AGAs (39). The initial results showed a promising activity of the combination, with an ORR of 77.6% (45/58, 5 pending confirmation), DCR was 100% and median PFS was not reached with 6-mo PFS rate of 84.6% for cohort 1B (Sac-TMT 5 mg/kg Q2W + KL-A167–900 mg Q2W) who had a median follow up of only 6.9 months (39). The results favored the combination in both the squamous and nonsquamous histology groups. A Phase III study of Sac-TMT Q2W plus pembrolizumab vs pembrolizumab in 1L metastatic NSCLC with PD-L1 TPS ≥ 50% (NCT06170788) is recruiting. A phase 1/2 trial of Sac-TMT alone (NCT04152499) in previously treated, advanced NSCLC with or without activating EGFR mutations showed a confirmed ORR 40% (17 of 43; 95% CI, 25-56), with better outcomes in the EGFR-mutant subset (22 of 43, 51%) with a confirmed ORR of 55% (12 of 22) from the *post-hoc* subgroup analyses (40) A phase II study of Sac-TMT alone in 64 pts with EGFR mutant NSCLC (NCT05631262) found that Sac-TMT led to confirmed ORR of 34% (22 of 64; 95% CI, 23–47) (40).

Clinical trials of Sac-TMT alone or in combination are now underway in different settings: for the treatment of stage III unresectable NSCLC, as an adjuvant after complete resection, or as first- or later-line treatment. Some of these trials are biomarker-agnostic, and others are biomarker-selected.

### 4.4 MET directed ADCs

C-Met protein overexpression occurs in approximately 20-25% of NSCLC patients, but it has not yet been clearly defined as a clinically useful biomarker (41, 42). MET immunohistochemistry (ICH) analysis is challenging due to substantial inter-laboratory variability, which may explain the lower efficacy of MET-targeted therapy in the c-Met-overexpressing patient population (42, 43).

Telisotuzumab vedotin (Teliso-V) consists of a humanized monoclonal antibody, telisotuzumab (ABT-700), coupled to the antimicrotubule payload MMAE through a valine-citrulline junction. In the phase II LUMINOSITY trial, Teliso-V was associated with durable responses in c-Met protein-overexpressing non-squamous EGFR-wild type previously treated NSCLC, especially in those with high c-Met expression (44). C-Met protein overexpression was defined as ≥25% tumor cells with 3+ staining (high [≥50% 3+]; intermediate [≥25%-<50%]). ORR was 34.6% [95% CI, 24.2 to 46.2] in the c-MET high group, 22.9% [95% CI, 14.4 to 33.4] in the c-MET intermediate group 28.6% (95% CI, 21.7 to 36.2 in the whole group of overexpressors (44). On May 14, 2025, the FDA granted accelerated approval to telisotuzumab vedotin-tllv for adults with locally advanced or metastatic NSCLC with high c-Met protein overexpression (≥50% of tumor cells with strong (3+) staining), as determined by an FDA-approved test, who have received a prior systemic therapy. The ongoing phase III TeliMET NSCLC-01 study compares Teliso-V at a dose of 1.9 mg/kg Q2W vs. docetaxel 75 mg/m^2^² Q3W in pts with locally advanced or metastatic c-Met-overexpressing EGFR WT non-squamous NSCLC who have progressed on prior therapy (45).

### 4.5 Targeting CEACAM5

CEACAM5 is a cell-surface glycoprotein expressed on epithelial cells of some solid tumors, including non-squamous NSCLC. In non-squamous NSCLC tumors, the prevalence of CEACAM5 high expression is 24.3%, defined as membranous CEACAM5 immunohistochemistry staining of ≥2+ intensity in ≥ 50% of tumor cells (46). Tusamitamab ravtansine (tusa rav), a CEACAM5-targeting ADC, was investigated in the CARMEN-LC03 (NCT04154956) Phase III open-label, randomized, pivotal, multicenter study evaluating tusa rav versus docetaxel in patients with advanced non-squamous NSCLC previously treated with platinum-based chemotherapy and immunotherapy (in combination or sequential), whose tumors highly expressed CEACAM5 (47). Tusa rav did not meet its dual primary endpoints of PFS and OS compared with docetaxel in patients with previously treated advanced non-squamous NSCLC; the median PFS was similar between the two groups, at 5.4 months with tusa rav and 5.9 months for docetaxel (hazard ratio [HR], 1.14; 95% CI, 0.86–1.51) (47). OS at the first interim analysis was also similar between the two study groups, at 12.8 months for tusa rav and 11.5 months for docetaxel (HR, 0.85; 95% CI, 0.64–1.11). In December 2023, the study was discontinued.

The number of potential new targets for ADCs in NSCLC over the last 5 years has been rapidly increasing, and as of November 2025, there are more than 20. Future strategies include combinations of ADCs with other molecules and with vaccines. There is a trend to investigate the ADCs in earlier stages of the disease, even in the neoadjuvant setting. Other new emerging classes of drugs are bi-specific ADCs and ADCs with dual payloads.

## 5 Toxicities of ADCs

Treatment-related toxicities of ADCs can be on- or off-target, and are influenced by: the tumor-associated antigen and its expression, the cytotoxic payload and its pharmacokinetics, the linker cleavage, the conjugation chemistry, the DAR, and several patient-related factors (age and organ function, comorbidities, body composition, ethnicity, etc.) (48, 49).

A systematic review and meta-analysis of Tang et al. found a clear relationship between toxicity and DAR, with higher G≥3 toxicity for any adverse events (AEs) (50). A total of 40 studies were included, a substantial number of which focused on ADCs for the treatment of different hematologic malignancies; nevertheless, a significant number of patients with solid tumors, mainly breast cancer, cervical cancer, gastric cancer, and urothelial cancer, treated with ADCs were included. Another important finding of the study was that a significantly higher rate of complex toxicities of higher grade was observed in patients treated with ADCs with cleavable linkers (47%) compared to those treated with ADCs with non-cleavable linkers (34%) (50). The authors speculated that treatment with ADCs with cleavable linkers leads to increased free payload concentration early in circulation and subsequently induces greater toxicity.

Most common AEs during ADC treatment are cytotoxic payload–related, including nausea, vomiting, alopecia, neutropenia, and lymphopenia (51). An extensive systematic review and meta-analysis by Zhu et al. included 169 clinical trials and 22,492 patients (51). It was found that the incidence of treatment-related adverse events (TRAEs) is high (91.2% (95% CI, 90.7%–91.7%; I^2^ = 95.9%) for all-grade AEs and 46.1% (95% CI, 45.2%–47.0%; I^2^ = 96.3%) for G≥3 AEs (51). The most common G≥3 AEs in this study were neutropenia, hypoesthesia, thrombocytopenia, febrile neutropenia, and lymphopenia (51).

The most recent systematic review and meta-analysis focusing on the safety of ADCs in lung cancer, to our knowledge, is that of He et al., which analyzes 28 studies with 3,127 participants (52). All included studies were conducted in patients with locally advanced, metastatic, or extensive-stage lung cancer, including 12 studies on small cell lung cancer and 18 on NSCLC (one adenocarcinoma study, two squamous cell carcinoma studies, and 15 NSCLC unspecified histology studies). Twenty-eight of the studies were analyzed quantitatively. The pooled incidence of all-grade TRAEs was 91.4%, while the pooled incidence of G≥ 3 TRAEs was 41.7% (52). An indirect comparison showed that compared to NSCLC, SCLC had worse safety outcomes, including all-cause mortality. Also, combination therapy had a less favorable safety profile than ADC monotherapy. The combination groups exhibited a higher incidence of toxicities and more high-grade toxicities compared to the monotherapy group, with higher all-cause mortality (52).

In this publication, as in previous studies, ADCs with cleavable linkers showed worse safety outcomes than those with non-cleavable linkers (52). Concerning G5 TEAEs, ADCs with cleavable linkers exhibited renal primarily and urinary disorders, whereas for ADCs with non-cleavable linkers, nervous disorders were the most common toxicity resulting in death (52).

Some significant toxicities are dependent on the cytotoxic payload class (49). For example, tubulin inhibitors like enfortumab vedotin and trastuzumab emtansine may cause thrombocytopenia, anemia, neutropenia, hepatic toxicity, and peripheral neuropathy, while topoisomerase inhibitors like sacituzumab govitecan and trastuzumab deruxtecan primarily cause gastrointestinal toxicity and neutropenia. Concerns regarding severe toxicity and potential long-term disabilities following cancer treatment may lead some patients to decline anticancer therapies despite medical recommendation (53).

Of note, using the same ADC for the treatment of different cancers may result in different toxicities (54).

### 5.1 Toxicity of ADCs in the treatment of ES-SCLC

The DLL-3 inhibitors, such as Rova-T, showed promising efficacy and manageable safety from phase I trials onward in the setting of ES-SCLC. Toxicity was expected with an ADC containing a cytotoxic payload, with the most common adverse events being thrombocytopenia, pleural and pericardial effusions, edema, photosensitivity, and skin reactions. The dose-limiting toxicity from the phase I trial was thrombocytopenia and liver function abnormalities, which led to the recommended dose of 0.3 mg/kg Q6W for the phase II trial. Of note, there was one fatal gastrointestinal bleeding reported in this trial (12). In the phase II TRINITY trial, the most severe G 3 AEs were again thrombocytopenia (11%), photosensitivity (7%), and pleural effusion (5%) (16). The phase III trial (TAHOE) sadly did not meet its primary endpoint and was considered negative, with 42% of TRAEs of G≥3 and 22% of fatal AEs, even though 9% of these were attributed to disease progression alone (14). Details on the latter are available in **Table 3**. Another DLL3 inhibitor, still named ZL-1310, was well tolerated in the phase Ia/Ib expansion cohort study, with G3 AEs or higher across all dose cohorts in 23% and serious TRAEs in 21%, the most common being anemia (11%) and neutropenia (13%). There were 5 cases of discontinuation due to TRAEs and 2 cases of G≥ 3 ILD; thus, a dose of < 2.0 mg/kg was chosen for ongoing phase II trials, since none of the serious TRAEs were observed at this dose level (19).

**Table 3:** Toxicity of ADCs in the treatment of ES-SCLC.

Trial	Mechanism of action	Phase	Drug and disease setting	Line setting	Any TRAE (ADC)	G 3–4 AE	G5 AE	TRAE that led to drug discontinuation
TAHOE (NCT03061812)	DLL3 inhibitors	III	Rovalpituzumab tesirine (Rova-T) vs. topotecan ES-SCLC	2^nd^ line	95% Pleural effusion 29% Decreased appetite 25% Dyspnea 25% Fatigue 25% Nausea 23% Pericardial effusion 20% Peripheral edema 18% Anemia 16% Photosensitivity 16% Thrombocytopenia 15%	42% Thrombocytopenia 9% Anemia 7% Dyspnea 7% Fatigue 5% Pleural effusion 4% Pneumonia 4% Neutropenia 3% Febrile neutropenia 1%	22% (Diesease progression 9%) Pneumonia 2% Asthenia 0.3% Dyspnea 0.3%	7% ILD Hematologic toxicity Fatigue Nausea Patient decision
NCT06179069		Ia/Ib	ZL-1310 < 2.0 mg/kg ES-SCLC	≥ 2nd line	NR	6%Anemia 2% Neutropenia 4%	0%	0%
IDeate-Lung01 (NCT05280470)	B7-H3 targeting ADCs	II	Ifinatumab Deruxtecan (I- DXd) 12 mg/kg ES-SCLC	2nd-3rd line	98% Nausea 50% Decreased appetite 43% Anemia 36% Neutropenia 33% Leukopenia 21% Asthenia 21% Infusion related reaction 14% Diarrhea 14% Fatigue 14% Vomiting 7%	50% Nausea 2% Decreased appetite 2% Anemia 12% Neutropenia 17% Leukopenia 7% Asthenia 0% Infusion related reaction 0% Diarrhea 5% Fatigue 5% Vomiting 2%	14% Septic shock 4% Disease progression 2% MOF 2% Pneumonia 2% Pneumocystis jirovecii 2% Pneumonia 2%	17% Pneumonia 9% pneumonitis/ILD 2% Pneumocystis jirovecii pneumonia 2% Radiation pneumonitis 2% Septic shock 2%
ARTEMIS-001 (NCT05276609)		Ia/Ib	HS-20093 All dose levels All solid tumors	≥ 2nd line	ND Leukopenia 90% Neutropenia 85% Anemia 82% Nausea 60% Lymphopenia 58% Pyrexia 58% Thrombocytopenia 53% Hypoalbuminemia 48% Asthenia 48% Vomiting 40% Infusion reaction 40% ↑AST 20% ↑bilirubin 20%	ND Leukopenia 42% Neutropenia 48% Anemia 27% Nausea 2% Lymphopenia 42% Pyrexia 0% Thrombocytopenia 28% Vomiting 10% ↑Bilirubin 2%	ND	6% Neutropenia 1 pt Febrile Neutropenia 1 pt Neutropenia + Thrombocytopenia 1 pt
NCT05434234		Ia/Ib	YL-201 All dose levels All solid tumors	≥ 2^nd^ line	37% Leukopenia 66% Anemia 64% Neutropenia 62% Lmphopenia 36% Thrombocytopenia 33% Anorexia 36% Nausea 26% Hypoalbuminemia 23% Alopecia 20% ↑AST/ALT 19% Vomiting 18% Fatigue 18% Pneumonia 11% ILD 1%	55% Neutropenia 32% Leukopenia 30% Anemia 25% Lmphopenia 20% Thrombocytopenia 14% Pneumonia 5% Anorexia 2% ILD 1% Nausea 1% Fatigue 1% ↑AST/ALT 1%	2.6%	5%
TROPiCS-03 (NCT03964727)	TROP-2 inhibitor	II	Sacituzumab Govitecan 10 mg/kg ES-SCLC	≥ 2nd line	100% Diarrhea 77% Fatigue 61% Neutropenia 56% Constipoation 42% Nausea 40% Alopecia 30% Anemia 30% Decreased appetite 23% Abdominal pain 19% Hypomagnesemia 16%	74% Neutropenia 44% Diarrhea 9% Hyponatriemia 7% Anemia 5% Stomatitis 5%	2% Neutropenic sepsis	0%
NCT05599984	SEZ6 inhibitors	I	ABBV – 706 3 mg/kg	≥ 2nd line	98% Anemia 60% Fatigue 66% Nausea 27% Vomiting 18%	77% Neutropenia 29% Anemia 27% Leukopenia 25%	13% None related to ABBV-706	4%
NCT03639194		I	ABV – 011 1 mg/kg expansion	≥ 2nd line	98% Fatigue 48% Nausea 45% Thrombocytopenia 40% Decreased appetite 38% Vomiting 35% Constipation 28% Hypokalemia 20% ↑ Bilirubin 18% ↑AST 15% ↑GGT Ascites 10%	65% Fatigue 10% Thrombocytopenia 10% Anemia 8% Hypokalemia 8% ↑ Bilirubin 3% ↑GGT 5%	18% None related to ABBV-011 (Disease progression, stroke, cardiac failure, respiratory failure)	13%

Drugs targeting the B7-H3 complex like ifinatumab deruxtecan (I-DXd) exhibit typical cytotoxic AEs like nausea, vomiting, hematologic adverse events and sometimes ILD, which is all generally well manageable, but did also lead to death in 14% and discontinuation of treatment in 17% of patients enrolled in this phase II trial, where especially the lung-related AEs were the most prominent and dangerous (24). After encouraging results, I-DXd at a higher dose of 12 mg/kg has been selected for a further phase III study in patients with relapsed SCLC following only one prior line of therapy (IDeate-Lung02; NCT06203210). Another B7-H3 inhibitor, HS-20093, showed encouraging results in the phase Ia/Ib ARTEMIS-001 trial, and the most common G3 TRAEs were neutropenia, leukopenia, lymphopenia, thrombocytopenia, and anemia across all dose levels (55). YL201, a novel B7H3-targeting ADC, also displayed TRAEs in 97% of patients, the most common being hematologic (leukopenia 66%, anemia 64%, neutropenia 61%), and amongst non-hematologic AEs, the most common were anorexia (35%), nausea (26%), and hypoalbuminemia (22%). G3 TRAEs occurred in 55% of patients, with neutropenia (32%) being the most frequent (26).

TROP-2 inhibitor sacituzumab govitecan (SG) maintained a consistent safety profile as previously reported, even in the pretreated ES-SCLC population that received both platinum-based chemotherapy and/or immune checkpoint inhibitors beforehand in the phase II basket trial (28). 43 ES-SCLC patients were included, with gastrointestinal and hematological TRAEs being the most common. However, none of them led to treatment discontinuation, and only one death resulted from neutropenic sepsis (28).

For the SEZ6 inhibitor named ABBV-706, the most common treatment-emergent adverse events (TEAEs) were anemia (51%), fatigue (41%), neutropenia (31%), and leukopenia (31%). G≥ 3 TEAEs occurred in 28 (57%) patients and were mainly hematologic: neutropenia (29%), anemia (27%), and leukopenia (25%). No pneumonitis or interstitial lung disease was observed. Gastrointestinal TEAEs (all G1/2) were seen in 55% of pts, with the most common being nausea (27%) and vomiting (18%). There were no treatment-related deaths, even though 13% of patients died during treatment (29). In the phase I trial with ABBV-011 for ES-SCLC, no MTD was identified, and the 2 mg/kg dose was selected for every 3-week administration. However, some late-onset hepatic toxicity (median onset 65 days) led to the reduction of the primary planned dose to 1.6 mg/kg in cycle 1 and 1.2 mg/kg in subsequent cycles. TEAEs leading to treatment discontinuation, dose interruption, or dose reduction occurred in 13%, 35%, and 15% of patients in the 1 mg/kg dose-expansion cohort, respectively. Over 97% of patients experienced at least one TEAE. The most common were fatigue (50%), nausea (42%), thrombocytopenia (41%), decreased appetite (40%), and vomiting (31%). G≥3 TEAE occurred in 64% of patients. Fatigue and thrombocytopenia (9% each) and increased GGT and pneumonia (7% each) were the most frequent. There were again no deaths related to the study drug (30).

### 5.2 Toxicity of the ADCs in the treatment of NSCLC

The first drug to enter the ADC arena for NSCLC was trastuzumab deruxtecan (T-DXd), which targets the mutant HER2 receptor. Initially, as seen from the breast cancer trials, interstitial lung disease was the dose-limiting toxicity; thus, the lower dose of 5.4 mg/kg Q3W was chosen for ongoing investigation based on the DESTINY-Lung02 trial (31). Details on the latter are available in **Table 4**. There, two doses of T-DXd were investigated, and patients in the higher-dose arm had almost 20% more G≥3 TRAEs. Among patients receiving T-DXd at 5.4 mg/kg, TRAEs led to discontinuation of T-DXd in 14% of patients, dose reduction in 17%, and interruption of T-DXd in 27%. TRAEs most commonly related to treatment discontinuation were ILD 6% and pneumonitis 5.0% in the 5.4 mg/kg arm, and also the only death attributable to TRAEs was ILD in one patient. Other AEs were mostly hematological and gastrointestinal and could be well managed with supportive therapy. Decreased left ventricular ejection fraction did not seem to be of specific concern in this trial (31).

**Table 4:** Toxicity of the ADCs in the treatment of NSCLC.

Trial	Mechanism of action	Phase	Drug and disease setting	Line setting	Any TRAE (ADC)	G 3–4 AE	G5 AE	TRAE that led to drug discontinuation
DESTINY – Lung02 Trial (NCT04644237)	Anti HER2	II	Trastuzumab Deruxtecan (T-DXd) 5.4 mg/kg HER2 positive NSCLC	≥ 2^nd^ line	96% Nausea 67% Fatigue 45% Neutropenia 43% Decreased appetite 40% Anemia 37% Constipation 37% Vomiting 32% Leukopenia 29% Thrombocytopenia 28% Diarrhea 23% Alopecia 22% ↑AST/ALT 22% ILD 13%	39% Neutropenia 19% Anemia 11% Fatigue 8% Thrombocytopenia 6% Leukopenia 5% Nausea 4% ↑AST/ALT 3% Vomiting 3% Decreased appetite 2% Diarrhea 1% ILD 1%	1% ILD	14%
HERTHENA – Lung02 Trial (NCT05338970)	Anti HER3	II	Patritumab Deruxtecan (HER3 – DXd) EGFR positive NSCLC	≥ 3^nd^ line (after EGFR TKI and platinum based chemotherapy)	ND Nausea 66% Thrombocytopenia 44% Decreased appetite 42% Neutropenia 36% Constipation 34% Anemia 33% Fatigue 31% Diarrhea 28% Vomiting 27% Leukopenia 26% Dyspnea 19% ↑AST/ALT 12-17% Hypokalemia 17%	65% Thrombocytopenia 21% Neutropenia 19% Leukopenia 10% Anemia 14% Fatigue 6% Hypokalemia 5% Dyspnea 4% Nausea 3% Decreased appetite 3% ↑AST 1%	2% Pneumonitis 1pt Pneumonia 1pt GI perforation 1pt Respiratory failure 1pt	7%
TROPION – Lung01 Trial (NCT04656652)	Anti TROP2	III	Dapotomab Deruxtecan (Dato-DXd) vs. Docetaxel	≥ 2^nd^ line	88% Stomatitis 48% Nausea 34% Alopecia 32% Decreased appetite 23% Asthenia 19% Anemia 15% Diarrhea 10% ILD 9% Neutropenia 5% Leukopenia 3%	76% Stomatitis 7% ILD 4% Anemia 4% Asthenia 3% Nausea 2% Neutropenia 1%	1% ILD/ pneumonitis 2pt Sepsis 1 pt	8% Pneumonitis 4% ILD 1%
EVOKE-01 (NCT05089734)		III	Sacituzumab Govitecan (SG) vs. Docetaxel	≥ 2^nd^ line	94% Fatigue 57% Diarrhea 53% Alopecia 53% Nausea 42% Anemia 40% Neutropenia 38% Constipation 29% Decreased appetite 26% Vomiting 21% Stomatitis 13% Leukopenia 13% Pruritus 13% Pyrexia 13% Febrile neutropenia 8%	67% Neutropenia 25% Fatigue 13% Pyrexia 13% Diarrhea 11% Febrile neutropenia 8% Anemia 6% Leukopenia 5% Decreased appetite 2% Vomiting 2% Stomatitis 1%	1% Febrile neutropenia 1pt Neutropenic colitis 1 pt Sepsis 1 pt Septic shock 1 pt	7%
NCT04152499		I/II	Sacituzumab Tirumotecan Dose expansion All solid tumors	≥ 2^nd^ line	93% Nausea 63% Alopecia 57% Anemia 50% Stomatitis 47% Vomiting 47% Neutropenia 43% Fatigue 37% Rash 37% Leukopenia 37% Thrombocytopenia 30% Decreased appetite 23%	57% Leukopenia 37% Anemia 27% Neutropenia 23% Thrombocytopenia 13% Rash 3%	0%	3% Pneumonitis 1 pt
NCT04152499 NCT05631262		I/II	Sacituzumab Tirumotecan For EGFR positive mNSCLC	≥ 2^nd^ line	98% Anemia 77% Leukopenia 68% Neutropenia 66% Stomatitis 62% Alopecia 47% Thrombocytopenia 35% Asthenia 26% Decreased weight 21% Nausea 19% Lymphopenia 19% Hypoalbuminemia 14%	ND Anemia 67% Thrombocytopenia 20% Febrile neutropenia 20% Stomatitis 7% Pneumonia 1%	0%	2%
LUMINOSITY Trial NCT03539536	Anti cMET	II	Telizotumab Vedozin (Teliso-V) Total NSCLC population	≥ 2^nd^ line	81% Peripheral neuropathy 30% Peripheral edema 16% Fatigue 14% Decreased appetite 11% ↑AST/ALT 10- 11% Pneumonitis 11% Hypoalbuminemia 11% Blurred vision 9% Keratitis 6%	28% Peripheral neuropathy 7% Fatigue 2% ↑ALT 4% Pneumonitis 3% Blurred vision 1%	2% ILD 3 pts	21% Peripheral Neuropathy ILD/Pneumonitis
CARMEN-LC03 trial NCT04154956	Anti CECAM5	III	Tusamitamab Ravtansine vs. docetaxel	≥ 2^nd^ line	73% Keratitis 26% Peripheral neuropathy 14% Neutropenia 4% Anemia 76% Thrombocytopenia 12% Asthenia 37% Diarrhea 23%	15% Keratitis 7% Neutropenia	ND	8%

For patritumab deruxtecan (HER3-DXd), most TRAEs were again hematological and gastrointestinal, with thrombocytopenia and neutropenia occurring in G≥ 3 in 21% and 19% of patients, respectively (32). There were four lethal TRAE cases, namely pneumonia, pneumonitis, GI perforation, and respiratory failure, one patient each. TRAEs were associated with dose interruption in 40% of patients, dose reduction in 21%, and treatment discontinuation in 7%. Five percent of patients were assumed to have ILD, of which one case was fatal (32). After promising results and a manageable toxicity profile, HER3-DXd is continuing to be studied in the phase III trial after osimertinib and platinum-based chemotherapy in patients with EGFR-positive NSCLC (56).

Anti-TROP2 drugs that entered the treatment arena for advanced NSCLC are Dato-DXd, SG, and sac-TMT. Some toxicities are similar, for example, fatigue, alopecia, hematological and gastrointestinal AEs, and infusion-related reactions (IRR). The incidence of IRRs for SG is reported at approximately 13% (all grades), with G3 or higher IRRs rare (<1%) (57). Most reactions are mild to moderate and manageable with premedication and supportive care (57). In TROPION-PanTumor01, among patients with NSCLC treated with Dato-DXd at 6.0 mg/kg, any-grade IRR occurred in 13 (26.0%) patients, and G3 IRR was observed in 1 (2%) patient (58). Both agents require vigilance for infusion-related reactions and premedication prior to any dose. Additionally, diarrhea, which occurs in up to 72% of patients treated with SG, should be closely monitored, as it may be due to early-onset cholinergic syndrome, and atropine should be readily available (59). Otherwise, patients treated with Dato-DXd experienced 26% of G 3 TRAEs, dose reductions in 20% and treatment discontinuation in 8% (33). Three patients (1%) treated with Dato-DXd had TRAEs leading to death: two cases of ILD/pneumonitis and one of sepsis (33). Additionally, patients experienced ocular toxicities with Dato-DXd, such as increased lacrimation (8%), dry eye (7%), and keratitis (4%), of which only 1% was G≥ 3 (33). For patients treated with SG, TRAEs leading to dose reductions occurred in 29%, and leading to treatment discontinuation in 7% (35). Since the SG payload, SN-38, is metabolized by the uridine diphosphate glucuronosyltransferase 1A1 (UGT1A1) enzyme, gene polymorphisms can result in reduced enzyme activity and increased toxicity; thus, identification of these patients is recommended and can drive dose–related decisions during treatment (60). The most common AEs with Sac-TMT were hematological and GI toxicities, with over 20% of patients experiencing febrile neutropenia (61). Of note, ILD was not common (1%), and none of the patients died due to severe TRAEs (40, 61).

The AE profile of Teliso-V is consistent with other cMET inhibitors, including peripheral sensory neuropathy, ocular toxicities (blurred vision and keratitis), and hypoalbuminemia. None of these TRAEs resulted in treatment discontinuation. Pneumonitis occurred in 11% of patients, and 3 cases of ILD were terminal (44).

The CEACAM5 inhibitor Tusamitamab ravtansine has a more favorable safety profile than docetaxel, with fewer high-grade or serious AEs, less treatment discontinuation, and fewer dose reductions (47). However, ocular toxicity (keratopathy/keratitis) is a distinct concern, affecting 1 in 4 patients and often requiring dose delay or modification (in 43%); 7% of G≥3 events occurred (47). Other TRAEs include cytopenias and peripheral neuropathy, which could be well managed. Dose reductions occurred in 17% of patients treated with tusa rav, and 8% discontinued treatment (47).

## 5 Discussion

ADCs represent a rapidly evolving therapeutic strategy in thoracic oncology, offering a bridge between targeted therapy and cytotoxic chemotherapy. In lung cancer, ADCs have already demonstrated significant clinical activity, particularly in biomarker-defined subsets of NSCLC. Nevertheless, the clinical benefit in SCLC remains investigational, with no regulatory approvals in Europe at present.

The clinical development of ADCs in NSCLC has accelerated following the success of trastuzumab deruxtecan (HER2-DXd), the first ADC approved by both the FDA and EMA for HER2-mutant disease. More recently, agents such as datopotamab deruxtecan (TROP2-directed) and telisotuzumab vedotin (c-MET-directed) have achieved accelerated FDA approval, further establishing ADCs as a significant therapeutic class. These advances highlight the importance of precise biomarker identification - HER2 mutations, EGFR resistance, c-MET overexpression, and HER3 or TROP2 expression - in selecting patients most likely to benefit. However, not all targets have translated into clinical success; for instance, CEACAM5-directed tusamitamab ravtansine failed to improve survival compared with docetaxel. This underlines the necessity for robust target validation and careful patient selection in ADC development.

In contrast, SCLC continues to pose unique biological and clinical challenges. Despite early enthusiasm for DLL3-targeting ADCs such as rovalpituzumab tesirine (Rova-T), subsequent phase III trials failed to demonstrate meaningful benefit, mainly due to off-target toxicity and limited durability of response. More recently, next-generation DLL3-directed ADCs (ZL-1310, SHR-4849) and agents targeting B7-H3 (ifinatamab deruxtecan, HS-20093, YL201) have shown encouraging response rates in early-phase studies, suggesting that improved linker technologies and optimized payloads may overcome prior limitations. Nevertheless, these remain early findings, and confirmatory evidence is awaited. Although ADCs have led to slightly better response rates and DOR than chemotherapy, it remains unclear whether they can replace platinum-based doublets at first- or second-line. Another significant point is that pretreated patients with SCLC are often in poor performance status and often have lots of co-morbidities, which implies a more careful assessment and consideration of potential adverse events from ADCs.

Safety continues to be a significant limitation in ADC therapy. Across studies, the overall incidence of treatment-related adverse events (TRAEs) exceeds 90%, with G ≥ 3 events occurring in roughly 40–45% of patients. Hematologic and gastrointestinal toxicities are most frequent, while interstitial lung disease (ILD) remains a distinct and potentially fatal complication, especially with deruxtecan-based payloads. ADC-related cytopenias may also compromise the delivery of subsequent therapies in heavily pretreated patients. Differences in linker stability, DAR, and payload mechanism contribute to variability in toxicity profiles. Notably, ADCs with cleavable linkers tend to cause higher systemic exposure and toxicity.

Treatment with ADCs requires careful baseline assessment and close monitoring, including regular evaluation of hematologic parameters, gastrointestinal toxicity, and early respiratory symptoms, given the risk of severe cytopenias and interstitial lung disease. Prompt recognition and management of toxicities, with dose interruptions, reductions, or permanent discontinuation when indicated, are essential to minimize morbidity. In frail patients - such as those with poor performance status (PS ≥2), underlying chronic obstructive pulmonary disease, or paraneoplastic syndromes - the risk -benefit ratio of ADC therapy warrants particular caution, as toxicity may be amplified and tolerability substantially reduced.

The therapeutic positioning of ADCs relative to chemotherapy remains to be fully defined. Most trials demonstrate higher response rates and longer response duration than docetaxel in second-line treatment, yet overall survival gains are modest or completely absent in some phase III studies. Whether ADCs can replace platinum-based doublets in first-line therapy is under active investigation, with several ongoing randomized trials exploring ADC–immunotherapy–chemotherapy combinations in metastatic and resectable NSCLC. The introduction of ADCs in the neoadjuvant and perioperative setting represents a promising but uncharted frontier, with ongoing phase I–II trials (e.g., NeoCOAST-2, MYTX-011) evaluating feasibility and pathological response endpoints.

Future development of antibody–drug conjugates is increasingly focused on novel design and treatment strategies aimed at improving efficacy and overcoming resistance. These include bispecific ADCs capable of targeting multiple tumor antigens, as well as dual-payload or advanced conjugation chemistries designed to enhance tumor cell killing while limiting systemic toxicity.

Another key area of uncertainty is drug resistance. So far, three main mechanisms for resistance have been identified: target antigen loss (like for example following long-term exposure to T-DM1 in HER2 positive breast cancer), altered ADC processing, and payload resistance (61). Beyond abnormal antigen expression levels, heterodimerization of the target antigen with other cell-surface receptors may contribute to resistance to ADCs (62). Of note, impaired payload efflux may lead to cross-resistance among ADCs sharing similar payloads (e.g., topoisomerase I inhibitors). Understanding these processes will be essential for guiding sequential ADC use and for designing rational combination regimens.

Finally, the future success of ADCs in lung cancer will depend on better biomarker strategies. Conventional IHC does not always correlate with response, and the optimal cut-off for antigen expression remains controversial. Multi-omic profiling, spatial transcriptomics, and analyses of circulating tumor DNA may provide more predictive tools. Patient-reported outcomes, quality-of-life measures, and the impact of ADC-related pneumonitis on radiotherapy or prior pulmonary disease also warrant careful evaluation, particularly in SCLC patients with poor performance status or paraneoplastic syndromes.

We have to admit that the review was not conducted as a formal systematic review and was limited to English-language publications, which may introduce selection and language bias. In addition, much of the available evidence—particularly in small-cell lung cancer—is derived from heterogeneous early-phase studies with limited follow-up, and the findings should therefore be interpreted cautiously.

In summary, ADCs have emerged as one of the most promising new drug classes in thoracic oncology. Their future integration into clinical practice will depend on optimizing safety, refining patient selection, and elucidating mechanisms of resistance. The next generation of ADCs - with improved linker chemistry, bispecific or dual-payload designs, and combination strategies holds the potential to transform the therapeutic landscape for both NSCLC and SCLC.

## 6 Conclusion

ADCs are promising new treatment options with the potential to be highly effective in different tumor types. The future of ADCs in lung cancer treatment depends on identifying efficacious combinations, reliable biomarkers of efficacy, and ways to manage their toxicities to maximize benefit for our patients.
